# Effects of Propofol versus Sevoflurane on Postoperative Pain and Neuroendocrine Stress Response in Oocyte Pickup Patients

**DOI:** 10.1155/2021/5517150

**Published:** 2021-04-10

**Authors:** Yavuz Orak, Fatma İnanç Tolun, Murat Bakacak, Aslı Yaylalı, Hakan Kıran, Hafize Öksüz, Adem Doğaner, Işıl Yağmur, Ahmet Altun

**Affiliations:** ^1^Department of Anesthesiology and Reanimation, Kahramanmaras Sutcu Imam University, Kahramanmaras, Turkey; ^2^Department of Biochemistry, Kahramanmaras Sutcu Imam University, Kahramanmaras, Turkey; ^3^Department of Obstetrics and Gynecology, Kahramanmaras Sutcu Imam University, Kahramanmaras, Turkey; ^4^Histology Department, Kahramanmaras Sutcu Imam University, Kahramanmaras, Turkey; ^5^Biostatistics and Medical Informatics, Kahramanmaras Sutcu Imam University, Kahramanmaras, Turkey; ^6^Department of Pharmacology, Sivas Cumhuriyet University, Sivas, Turkey

## Abstract

**Background:**

Pain aggravates the autonomic response to stress and raises neuroendocrine stress hormone levels. We compared the effects of propofol and sevoflurane on postoperative pain and neuroendocrine stress hormones. A prospective, randomized, and controlled trial was conducted with 60 patients.

**Methods:**

We randomly allocated patients to groups P (remifentanil/propofol, *n* = 30) and S (remifentanil/sevoflurane, *n* = 30). Preoperative blood samples were taken to measure serum adrenocorticotropic hormone (ACTH), corticotropin-releasing hormone (CRH), glucagon, cortisol, aldosterone, and prostaglandin E2 (PGE2) levels. Intraoperatively and postoperatively, clinical parameters were monitored at different time points. The hormone levels were again measured in the follicular fluid and blood postoperatively.

**Result:**

Demographic data were similar. The preoperative serum aldosterone levels were significantly higher in group P (*p*=0.001). Preoperative and postoperative serum ACTH, glucagon, cortisol, and PGE2 levels were significantly different in group P (*p*=0.009, *p*=0.004, *p*=0.029, and *p*=0.002); serum ACTH, glucagon, and PGE2 levels increased while serum cortisol levels decreased postoperatively. In group S, serum CRH and aldosterone levels, both increased in the postoperative period compared to the preoperative (*p*=0.001, *p*=0.006). Postoperatively, glucagon and PGE2 levels were both higher in group P than group S (*p*=0.019, *p*=0.015). In postoperative follicular fluid, glucagon and PGE2 levels were higher in group P, while cortisol levels were higher in group S (*p*=0.001, *p*=0.007, and *p*=0.001).

**Conclusion:**

The effects of anesthetic agents were different. In group P, in the preoperative and postoperative evaluation, ACTH, glucagon, and PGE2 increased postoperatively, while cortisol decreased. In group S, aldosterone and CRH increased postoperatively. Glucagon and PG E2 were higher in group P than S, postoperatively.

## 1. Introduction

*In vitro* fertilization (IVF) techniques include the following steps [1]:

1. Ovarian stimulation: Medication is administered to the ovaries to produce eggs.
2. Egg collection (follicular aspiration): Ultrasound-guided transvaginal oocyte retrieval is performed to remove eggs from the woman's body.
3. Fertilization: Top-quality eggs and sperm are stored in a suitable room. The sperm is placed into the eggs. This process is called fertilization.
4. Embryo culture: The fertilized egg is divided into an embryo.
5. Embryo transfer into the uterus: The embryo is inserted into the uterus 3–5 days after the follicular aspiration.

Oocyte retrieval is a short procedure, usually lasting for 20 ± 30 minutes. It is carried out under an ultrasonography-guided transvaginal approach. To achieve this, it is necessary to consider a fast-acting anesthetic agent with minimum side effects [2]. Follicular fluid (FF) is a liquid mainly composed of enzymes, hormones, electrolytes, anticoagulants, reactive oxygen species, and antioxidants. FF is a crucial element for the performance of natural fertilization, existent at every stage of the conception process [3].

Propofol is a fast-acting, intravenous (iv) anesthetic agent and is commonly used for oocyte retrieval [4]. Remifentanil is a potent analgesic and selective opioid receptor agonist. Remifentanil's initial effect occurs in about 1 min, and it quickly reaches a consistent status. Remifentanil is metabolized with nonspecific blood and tissue esterases [5]. Sevoflurane is a volatile anesthetic agent used for the induction and maintenance of general anesthesia. It has several advantages in obstetrics: it has a low blood/gas partition coefficient in blood concentration, it is rapidly eliminated from the newborn after birth, and the patient quickly recovers from general anesthesia [6]. Puncturing of both vaginal skin and the ovarian capsule to aspirate oocytes causes discomfort similar to severe menstrual pain [7]. Pain aggravates the autonomic stress response and also raises stress hormone levels [8].

In this study, we compared the effects of propofol and sevoflurane on pain and adrenocorticotropic hormone (ACTH), corticotropin-releasing hormone (CRH), glucagon, cortisol, aldosterone, prostaglandin E2 (PGE2) levels in the blood and follicle fluid in oocyte pick-up (OPU) patients.

## 2. Materials and Methods

### 2.1. Patients

This prospective, randomized study was conducted between May 2018 and April 2019 at the IVF center of the Department of Obstetrics and Gynecology, Faculty of Medicine, Kahramanmaras Sutcu Imam University, Turkey. The ethics committee approved the study (decision number: 2017/12–16 and date: July 19, 2017) and was registered with the United States National Clinical Trials Registry (Clinical Trials Number: NCT03507621). All patients provided written informed consent.

The patients were aged between 18 and 40 years and, with the American Society of Anesthesiologists (ASA) physical status 1, were included in the study. The patients with psychological, cardiac, renal, and liver disorders, those <18 or >40 years old, those with drug allergies, and those unwilling to participate in the study were excluded. Finally, 60 patients were divided into two study groups: groups P (remifentanil/propofol, *n* = 30) and S (remifentanil/sevoflurane, *n* = 30). Before the patients entered the operating room, sealed opaque envelopes were handed to the patients for their group assignment in the patients' room. Patients were randomized into groups with a 1 : 1 ratio. The participants were allocated to the groups as per the random allocation order.

Each patient's age and body mass index (BMI) were recorded preoperatively. Preoperative blood samples were taken from patients to measure serum ACTH, CRH, glucagon, cortisol, aldosterone, and PGE2 levels. Blood and follicle samples were taken from all patients between 8.30 and 11.00 a.m. Samples were taken in a yellow capped 13 × 100 5 mL BD vacutainer plastic SST gel tube (Becton Dickinson, NJ, USA). Adrenocorticotropic hormone (ACTH) and aldosterone/renin samples must be transported rapidly to the laboratory for analysis [9]. A study showed that ACTH and aldosterone/renin samples could be studied for 6 hours at room temperature when taken under appropriate conditions [10]. In this study, all the samples were taken to the laboratory without waiting, centrifuged at –4°C, and stored at −80°C until analysis. Group P was administered iv remifentanil at a dose of 0.5 mcg/kg over 30 sec (Ultiva 2 mg vial, Glaxo Smith Kline, UK) and iv propofol at a dose of 1 mg/kg (1% ampoule, Fresenius Kabi, Germany). In contrast, group S was administered iv remifentanil at a dose of 0.5 mcg/kg over 30 sec and sevoflurane 3–5 % (Sevorane R, Abbvie, Istanbul, Turkey) via inhalation. Additional analgesia with remifentanil and additional anesthesia with sevoflurane and propofol were administered according to the patient's body movements. During the operation, systolic blood pressure (SBP), diastolic blood pressure (DBP), mean arterial pressure (MAP), heart rate (HR), and SpO2 were recorded at 1, 3, 5, 7, 10, and 15 minutes. After the end of the operation, hemodynamic parameters were recorded. Hormone levels in the blood and follicular fluid were measured postoperatively.

### 2.2. Score Measurements

The depth of anesthesia was monitored using a graded intraoperative movement (IOM) scale from 0 to 4. IOMs recorded during surgery were considered a reaction to nociceptive stimulation. Analgesia and/or anesthesia was administered based on these IOMs [11]:

- **Grade 0:** no movement
- **Grade 1:** ankle movements (could deepen analgesia)
- **Grade 2:** knee movements (could deepen analgesia/anesthesia)
- **Grade 3:** pelvic and hip movements (analgesia/anesthesia must be deepened)
- **Grade 4:** rude pelvic, chest, and/or arm movements (anesthesia must be deepened)

Before the operation, the visual analog scale (VAS) score for pain was explained to all patients (pain severity: 0 indicated no pain, while 10 indicated maximum pain). In the postoperative period, each patient's pain was evaluated using the VAS score at 1, 5, 15, 30, and 60 minutes after the patient regained consciousness. If a patient had a VAS score ≥5 or wanted a painkiller, 75 mg of diclofenac sodium was administered intramuscularly.

### 2.3. Ovarian Stimulation

After routine infertility tests, ovulation induction, by gonadotropin-releasing hormone antagonist protocol, was started in all patients on the second or third day of the menstrual cycle. Human chorionic gonadotropin (Ovitrelle, Merck Serono, Modugno, Italy) was made (250 mcg/0.5 mL) on day one, follicle >17 mm was observed in follicle measurements, and eggs were collected at 36 h (OPU). All the procedures performed in the routine embryology laboratory were recorded.

### 2.4. Biochemical Analysis

An automatic enzyme-linked immunosorbent assay (ELISA) microplate reader (Thermo Fisher Scientific, Finland) and a computer program (Skanlt for Multiscan FC 2.5.1) were used.

#### 2.4.1. ACTH

Serum ACTH levels were determined with ELISA using a commercial kit (USCN Business Co., Ltd.). The sensitivity was 0.7 pg/mL, the detection range was 1.6–1000 pg/mL, the intra-assay coefficient of variation (CV) was <10%, and the interassay CV was <12%. Results were observed as pg/mL.

#### 2.4.2. CRH

Serum CRH levels were determined with ELISA using a commercial kit (USCN Business Co, Ltd). The sensitivity was <5.19 pg/mL, the detection range was 12.35–1000 pg/mL, the intra-assay CV was <10%, and the interassay CV was <12%. Results were observed as pg/mL.

#### 2.4.3. Glucagon

Serum glucagon levels were determined by ELISA using a commercial kit (USCN Business Co, Ltd). The sensitivity was <0.7 pg/mL, the detection range was 1.6–1000 pg/mL, the intra-assay CV was <10%, and the interassay CV was <12%. Results were observed as pg/mL.

#### 2.4.4. Cortisol

Serum cortisol levels were determined by ELISA using a commercial kit (Diametra, Assisi, Italy). The sensitivity was 2.42 ng/mL, the intra-assay CV was ≤5.1%, and the interassay CV was ≤11.0%. Results were observed as ng/mL.

#### 2.4.5. Aldosterone

Serum aldosterone levels were determined by ELISA using a commercial kit (Diametra). The intra-assay CV was <9.7%, and the interassay CV was <11%. Results were observed as pg/mL.

#### 2.4.6. PGE2

Serum PGE2 levels were determined by ELISA using a commercial ELISA kit (Shanghai Sunred Biology Technology Co. Ltd., China). The sensitivity was 2,423 ng/L, and the analysis range (assay range) was 3 ng/L–900 ng/L. Results were observed as ng/mL.

### 2.5. Statistical Analysis

Power analysis was used to determine the sample size of the study. The reference study was based on postoperative pain scores [12]. Our study included two groups and VAS, and neuroendocrine stress parameters were primary outcome measures. Hemodynamic parameters and oxygen saturation were secondary outcome measures. According to the reference study's statistical parameters, the following were the groups' values: group 1; 2 (2.8), group 2; 5 (5), alpha = 0.05 first type error level, and beta = 0.20-second type error level, at 0.80 test power. A total of 60 patients were included in the study (*n* = 30 for each).

The variables' suitability for normal distribution was checked using the Shapiro–Wilk test in data evaluation. Normally distributed variables and comparison groups were analyzed using the independent sample *t*-test. Preoperative and postoperative differences were examined using the paired *t*-test. In nonnormally distributed variables, comparison groups were analyzed using the Mann–Whitney *U* test. Statistical parameters were expressed as mean ± standard deviation (median 25% quartile to 75% quartile). The relationship between the distribution of categorical variables was analyzed using the chi-square test and Fisher's exact test. Pearson correlation test was used for correlation analysis. *p* < 0.05 was considered statistically significant. Data were analyzed using SPSS for Windows version 22 (IBM Corporation, NY, USA).

## 3. Results

**Figure 1:**
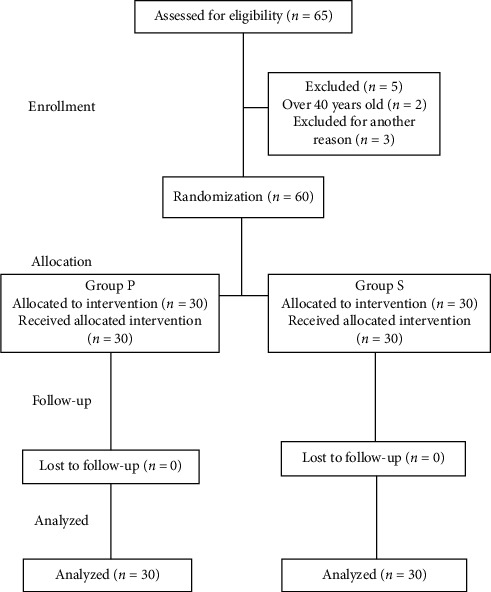
The CONSORT flow diagram of groups. CONSORT: Consolidated Standards of Reporting Trials. Group P: propofol + remifentanil. Group S: sevoflurane + remifentanil.

A total of 60 patients were included in this study.  Figure 1 shows the flow diagram of forming groups in this study. We found no difference between the two groups in terms of age, BMI, and operation time. Remifentanil requirements of the patients were higher in group P compared to group S (*p* < 0.001). There was no statistically significant difference between the two groups in terms of postoperative VAS scores and postoperative analgesic requirements (Table 1).

The preoperative serum aldosterone levels were significantly different between groups P and S, higher in group P (*p*=0.001). Preoperative and postoperative serum ACTH, glucagon, cortisol, and PGE2 levels were significantly different in group P (*p*=0.009, *p*=0.004, *p*=0.029, and *p*=0.002); serum ACTH, glucagon, and PGE2 levels increased while serum cortisol levels decreased postoperatively. We found a significant difference between preoperative and postoperative serum CRH and aldosterone levels in group S (*p*=0.001, *p*=0.006); both increased postoperatively. Glucagon and PG E2 levels were significantly different between groups in postoperative serum measurements (*p*=0.019, *p*=0.015), glucagon, and PGE2 levels were higher in group P (Table 2). Glucagon, cortisol, and PGE2 levels were significantly different between groups in postoperative follicular fluid measurements (*p*=0.001, *p*=0.007, *p*=0.001). Glucagon and PGE2 levels were higher in group P. In contrast, cortisol levels were higher in group S (Table 3).

In the preoperative period, the negative correlation between the level of glucagon and the level of CRH in group P was found to be significant (*r*: −0.402 *p*: 0.027). While the level of glucagon increased, the level of CRH decreased. No statistically significant difference was observed among the relationships in other variables. In Group S, no relationship was detected among variables in terms of preoperative values (Table 4).

In the postoperative period, no relationship was found among the variables in group P. In group S, a statistically significant positive correlation was found between the level of CRH and the level of cortisol (*r*: 0.429 *p*=0.018). While the level of CRH increased, the level of cortisol increased. No other relationship was observed between other variables (Table 5).

In terms of postoperative follicle levels, the positive correlation between the level of glucagon and the level of cortisol in group P was found to have a statistically significant difference, respectively (*r*: 0.507 *p*: 0.004; *r* = −0.485 *p*: 0.007). While the level of cortisol increased, the level of glucagon also increased. In group S, a positive correlation was found between the level of CRH and the level of glucagon (*r*: 0.505 *p*: 0.007). While the level of CRH increased, also the level of glucagon increased (Table 6).

In group P, IOMs were found to be higher in each measurement (*p*=0.001, *p*=0.001, *p*=0.001, *p*=0.003, *p*=0.002) (Table 7).

**Figure 2:**
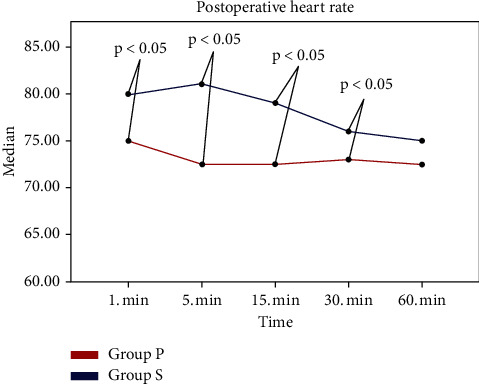
Between-group comparison of the HR postoperatively. HR: heart rate.

During the intraoperative period, SpO2 was significantly lower in group P at 1, 3, 5, 7, 10, and 15 minutes compared to group S (*p*=0.001, *p* ≤ 0.001, *p* ≤ 0.001, *p* ≤ 0.001, *p*=0.001, and *p*=0.002). Postoperatively, the HR was significantly lower in group P at 1, 5, 15, and 30 minutes compared to group S (*p*=0.042, *p*=0.008, *p*=0.004, and *p*=0.049) (Figure 2).

## 4. Discussion

In the present study, propofol and sevoflurane had different effects on ACTH, CRH, glucagon, cortisol, aldosterone, and PGE2 levels in the blood and follicular fluid. Glucagon and PGE2 were higher in group P, while cortisol was higher in group S in postoperative follicular fluid. Serum ACTH, glucagon, and PGE2 increased, while serum cortisol decreased postoperatively in group P. Serum CRH and aldosterone increased, postoperatively in group S.

A study on mice showed that sevoflurane promotes PG E2 production in peritoneal macrophages [13]. Other studies have reported that propofol improves ear edema formation and reduces PGE2 production [14], inhibiting cyclooxygenase activity, and suppressing PG E2 production from dendritic cells [15]. Considering PG E2 regulates fertilization and protects the sperm against phagocytosis in the ovary [16], propofol came to the forefront in our study about PGE2. In our study, PG E2 levels were higher in serum and follicular fluid in group P than group S postoperatively. In serum, PG E2 also increased significantly in the postoperative period compared to the preoperative period in group P. PG E2 has a significant effect on the processing of pain signals [17]. In group P, the patients may have felt more pain during the intraoperative period because a higher dose of remifentanil was used. One study revealed that dose-dependent acute injections of fentanyl increased spinal cyclooxygenase-2 (COX-2) mRNA and PGE2 protein levels in rats [18]. Another study conducted on humans showed that acute remifentanil infusion did not enhance the level of cerebrospinal fluid PGE2 [19]. These two different studies were conducted in rats and humans [18, 19], and different doses were used. In this study, in group P, the increase in PG E2 in serum and follicular fluid may be due to also the use of higher doses of remifentanil and pain in addition to propofol use. Daş et al. [20] found that propofol had no effects on glucagon levels in a study carried out on an adult pig. In contrast, a study on lower abdominal surgeries found that glucagon levels are higher in the propofol/sufentanil group at 2 hours postoperatively compared with the preoperative levels [21]. Similarly, in this study, the postoperative glucagon level in group P increased compared to the preoperative level. In group P, both follicle and postoperative serum glucagon levels were found higher than group S.

One study on rats has shown that sevoflurane increased CRH messenger RNA levels in the hypothalamus for 60 min [22], which is consistent with the findings of our study. The stress response to surgery causes hormonal change initiated by the neuronal activation of the hypothalamic–pituitary–adrenal axis, and CRH level increases in stress response to surgery. CRH increases ACTH and cortisol release. Besides, it increases the release of catecholamines, glucagon, prolactin, growth hormone, and b-endorphin [23]. In correlation analysis, glucagon increases as cortisol increases in postoperative follicular fluid in group P, and glucagon increases as CRH increases in group S, which is compatible with the literature. The increase in ACTH in plasma, followed by the increase in cortisol, is related to the severity of the surgical injury [24]. The correlation between the increase in CRH and cortisol in the serum in the postoperative period in Group S is compatible with the literature. The use of sevoflurane anesthesia in the low-stress laparoscopic procedure significantly reduces ACTH and cortisol [25]. Harlow et al. [26] reported that patients' anxiety and cortisol levels increase during IVF treatment. Studies have shown that high anxiety and cortisol levels are associated with low pregnancy rates after IVF, and low follicular cortisone levels and high cortisol/cortisone ratio have especially significant effects on the pregnancy rate [27, 28]. Acar et al. [29] reported that cortisol levels at 1 h are lower in the propofol group than in the sevoflurane and spinal anesthesia groups. Chen et al. [30] showed that stress hormone levels are lower in the propofol group than in the sevoflurane group. Propofol infusion completely abolished cortisol secretion under deep anesthesia [31]. These findings indicated that propofol suppresses the stress response better, which is consistent with the findings of our study. In this study, propofol and remifentanil were administered to the patients according to body movements during the operation. Group P also needed more remifentanil during the operation. Remifentanil may have decreased the cortisol level in serum and follicular fluid. Because there are studies showing that remifentanil reduces the cortisol level in addition to propofol [32, 33], in laparoscopic colectomy, continuous remifentanil infusion suppressed the responses of the hypothalamic–pituitary–adrenal axis [34]. In this study, the postoperative cortisol level in group P was lower than the preoperative level and follicle cortisol level was lower in group P compared to group S. In group P, increased ACTH and decreased cortisol without increasing CRH is a contradiction. The operation did not involve a surgical incision, its duration, and its difference from other studies may have affected our results. In the previous study, which showed the presence of mineralocorticoids in human ovarian follicles, aldosterone, and precursor corticosterone levels in the follicular fluid were significantly high. In contrast, aldosterone levels in the follicular fluid were significantly high compared with those in the blood [35], which is consistent with the findings of this study. In this study, the follicular aldosterone level was approximately three times larger than the serum aldosterone level. One study on percutaneous nephrolithotomy patients reported that renin, aldosterone, and ACTH levels were significantly high in the sevoflurane group at 15 minutes after irrigation compared with the total intravenous anesthesia group [36]. A study showed that sevoflurane increases serum aldosterone levels [37]. In this study, the blood level of aldosterone in group S was lower preoperatively than group P. Therefore, we were suspicious about the preoperative and postoperative significant increase in group S.

In a study of day surgery patients anesthetized, patients felt less pain in the propofol group than in the sevoflurane group [38]. In patients undergoing gynecological procedures, the effects of sevoflurane, desflurane, and propofol anesthesia on postoperative morphine consumption and pain were not different [39]. No difference was between groups in terms of postoperative 1^st^ hour VAS scores in the present study. Higher remifentanil requirements of patients in group P during operation may have affected postoperative VAS scores. Although intraoperative SpO2 and postoperative HR values were lower with propofol + remifentanil, these values, including other hemodynamic parameters, were within the clinically normal range. In group P, more remifentanil was consumed as the IOMs were higher; however, intraoperative HR did not differ.

Limitations: first, anxiety scores were not measured in this study and, second, the bispectral index score was not used to measure the depth of anesthesia.

## 5. Conclusion

Our study was the first to show PGE2, CRH, aldosterone, and glucagon levels in the blood and follicular fluid in OPU patients. Postoperative blood and follicular fluid cortisol levels were lower with propofol + remifentanil. Considering the regulation of PGE2 fertilization with propofol + remifentanil, and the protective effect of sperm against phagocytosis in the ovaries, higher PGE2 levels in follicular fluid with propofol + remifentanil, compared to sevoflurane + remifentanil, show that propofol is more effective. In the correlation analysis, in group P, we did not care clinically about the increase in glucagon while CRH decreased in the preoperative period since it was a preoperative measured value. Other correlation analyses made our study valuable.

## Figures and Tables

**Table 1:** Sociodemographic and clinical data of patients.

		Group P (*n* = 30)	Group S (*n* = 30)	Statistics	*p*
Age	Mean ± SD	31,87 ± 5,26	33,97 ± 5,10	1,570^a^	0,122
BMI	Mean ± SD	25,81 ± 3,59	26,55 ± 4,75	0,678^a^	0,501
Operation time	Median (Q1–Q3)	10,00 (10,00–15,00)	10,00 (7,00–15,00)	414,500^b^	0,579
PAAR	Median (Q1–Q3)	2,00 (2,00–2,00)	2,00 (1,00–2,00)	416,500^b^	0,710
IRD	Median (Q1–Q3)	35,00 (30,00–40,00)	35,00 (30,00–40,00)	412,500^b^	0,567
IPD	Median (Q1–Q3)	70,00 (60,00–80,00)			
ARRDO	Median (Q1–Q3)	20,00 (20,00–40,00)	0,000,00–0,00)	167,000^b^	*p* < 0.001^*∗*^
APRDO	Median (Q1 Q3)	60,00 (40,00 80,00)			
VAS 1^st^.dk	Median (Q1–Q3)	2,00 (1,00–4,00)	2,00 (0,00–3,00)	322,500b	0,054
VAS 5^th^ min	Median (Q1–Q3)	2,00 (1,00–4,00)	2,00 (0,00–4,00)	369,000^b^	0,224
VAS 15^th^ min	Median (Q1–Q3)	2,00 (1,00–4,00)	2,00 (0,00–3,00)	359,000^b^	0,171
VAS 30^th^ min	Median (Q1–Q3)	2,00 (1,00–5,00)	2,00 (1,00–4,00)	406,500^b^	0,661
VAS 60^th^ min	Median (Q1–Q3)	2,00 (1,00–3,00)	2,00 (0,00–4,00)	416,000^b^	0,609

^a^Independent samples *t*-test. ^b^Mann–Whitney *U* test. *α* = .05. ^*∗*^ Statistically significant difference. Postoperative additional analgesic requirement: PAAR. Additional remifentanil requirement during operation: ARRDO. Additional propofol requirement during operation: APRDO. Induction remifentanil dose: IRD. Induction propofol dose: IPD.

**Table 2:** Between-group and intragroup comparisons of blood parameters between groups P and S.

		Group P	Group S	*p* values
		(*n* = 30)	(*n* = 30)	
ACTH (pg/mL)	Preoperative	136,15 ± 8,11	137,93 ± 11,35	0,487
	Postoperative	140,81 ± 6,85	138,55 ± 11,52	0,359
	P^a^	**0,009** ^*∗*^	0,859	

Aldosterone (pg/mL)	Preoperative	37,98 ± 16,14	23,78 ± 15,75	**0,001** ^*∗*^
	Postoperative	37,94 ± 14,30	36,05 ± 18,79	0,663
	P^a^	0,985	**0,006** ^*∗*^	

CRH (pg/mL)	Preoperative	11,89 ± 1,51	11,25 ± 1,18	0,073
	Postoperative	11,93 ± 1,01	12,23 ± 1,01	0,254
	P^a^	0,884	**0,001** ^*∗*^	

Glucagon (pg/mL)	Preoperative	104,52 ± 4,92	104,79 ± 18,77	0,940
	Postoperative	107,50 ± 3,93	103,60 ± 7,89	**0,019** ^*∗*^
	P^a^	**0,004** ^*∗*^	0,761	

Cortisol (ng/mL)	Preoperative	146,02 ± 32,63	137,15 ± 35,77	0,320
	Postoperative	129,20 ± 35,45	138,07 ± 39,74	0,365
	P^a^	**0,029** ^*∗*^	0,924	

PG E2 (ng/mL)	Preoperative	34,67(30,65–51,68)	33,99(32,35–37,43)	0,802^b^
	Postoperative	42,50(34,17–102,87)	33,50(31,15–40,83)	**0,015** ^*∗*^^**b**^
	P^c^	**0,002** ^*∗*^	0,644	

Independent samples *t*-test; ^a^paired *t*-test; ^b^Mann–Whitney *U* test; ^c^Wilcoxon test; a: 0.05; ^*∗*^statistically significant. ACTH, adrenocorticotropic hormone; CRH, corticotropin-releasing hormone; and PGE2, prostaglandin E2.

**Table 3:** Comparison of follicular fluid levels between groups P and S.

Follicle	Group P (*n* = 30)	Group S (*n* = 30)	*p*
ACTH (pg/mL)	137,76 ± 14,26	142,39 ± 20,94	0,329
Aldosterone (pg/mL)	84,91 ± 19,48	88,67 ± 9,09	0,363
CRH (pg/mL)	10,85 ± 2,38	11,79 ± 1,38	0,078
Glucagon (pg/mL)	99,53 ± 6,88	87,37 ± 15,99	**0,001** ^*∗*^
Cortisol (ng/mL)	92,49 ± 44,23	122,31 ± 35,68	**0,007** ^*∗*^
PGE 2 (ng/mL)	69,52 (46,59–156,84)	32,74 (26,71–39,18)	**0,001** ^*∗*^^**a**^

Independent samples *t*-test; ^a^Mann–Whitney *U* test; *α*: 0.05; ^*∗*^statistically significant. ACTH, adrenocorticotropic hormone; CRH, corticotropin-releasing hormone; and PGE2, prostaglandin E2.

**Table 4:** Preoperative correlation analysis of blood variables between groups.

		ACTH (pg/mL)		CRH (pg/mL)		Glucagon (pg/mL)	
Preoperative correlation		r	p	r	p	r	p
Group P	CRH (pg/mL)	−0,322	0,082				
	Glucagon (pg/mL)	0,269	0,150	−0,402	**0,027** ^*∗*^		
	Cortisol (ng/mL)	0,105	0,580	−0,211	0,262	0,134	0,480

Group S	CRH (pg/mL)	0,017	0,927				
	Glucagon (pg/mL)	−0,069	0,716	−0,155	0,414		
	Cortisol (ng/mL)	0,336	0,069	0,067	0,724	0,145	0,445

^a^Pearson correlation test; *α*: 0.05. ^*∗*^Statistical significance. CRH: corticotropin-releasing hormone and ACTH: adrenocorticotropic hormone.

**Table 5:** Postoperative correlation analysis of blood variables between groups.

		ACTH (pg/mL)		CRH (pg/mL)		Glucagon (pg/mL)	
Postoperative correlation		r	p	r	p	r	p
Group P	CRH (pg/mL)	−0,084	0,659				
	Glucagon (pg/mL)	0,317	0,088	−0,014	0,942		
	Cortisol (ng/mL)	−0,021	0,911	0,061	0,748	−0,023	0,905

Group S	CRH (pg/mL)	−0,174	0,357				
	Glucagon (pg/mL)	0,067	0,727	−0,023	0,905		
	Cortisol (ng/mL)	0,149	0,433	0,429	**0,018** ^*∗*^	−0,080	0,673

Pearson correlation test; *α*:0.05. ^*∗*^Statistical significance. CRH: corticotropin-releasing hormone and ACTH: adrenocorticotropic hormone.

**Table 6:** Correlation analysis of postoperative follicular fluids between groups.

Follicular fluid		ACTH (pg/mL)		CRH (pg/mL)		Glucagon (pg/mL)	
		r	p	r	p	r	p
Group P	CRH (pg/mL)	0,248	0,187				
	Glucagon (pg/mL)	0,243	0,196	0,161	0,396		
	Cortisol (ng/mL)	0,337	0,069	0,114	0,550	0,507	**0,004** ^*∗*^

Group S	CRH (pg/mL)	−0,024	0,906				
	Glucagon (pg/mL)	0,060	0,766	0,505	**0,007** ^*∗*^		
	Cortisol (ng/mL)	−0,064	0,750	-0,291	0,140	-0,028	0,891

Pearson correlation test; *α*: 0.05. ^*∗*^Statistical significance. CRH: corticotropin-releasing hormone and ACTH: adrenocorticotropic hormone.

**Table 7:** Evaluation of intraoperative movements.

		Group P			Group S		
		*n* = 30	%	*n* = 30	%	*X* ^2^	*p*
Grade 0	No	8	26,7	25	83,3	19,461	**0,001** ^*∗*^
	Yes	22	73,3	5	16,7		

Grade 1	No	10	33,3	26	86,7	17,778	**0,001** ^*∗*^
	Yes	20	66,7	4	13,3		

Grade 2	No	14	46,7	26	86,7	10,800	**0,001** ^*∗*^
	Yes	16	53,3	4	13,3		

Grade 3	No	15	53,6	27	93,1	11,557	**0,003** ^*∗*^
	Yes	13	46,4	2	6,9		

Grade 4	No	15	50	26	86,7	9,319	**0,002** ^*∗*^
	Yes	15	50	4	13,3		

Chi-square test, Fisher's exact test,*α* = .05. ^*∗*^Significant relationship between groups. No: body movement none. Yes: there is body movement.

## Data Availability

The data used to support the findings of this study are available from the corresponding author upon request.
